# Mitochondrial genomes assembled from non-invasive eDNA metagenomic scat samples in the endangered Amur tiger *Panthera tigris altaica*

**DOI:** 10.7717/peerj.14428

**Published:** 2022-12-06

**Authors:** J. Antonio Baeza

**Affiliations:** 1 Department of Biological Sciences, Clemson University, Clemson, SC, United States; 2 Smithsonian Marine Station at Fort Pierce, Fort Pierce, Florida, United States; 3 Departamento de Biologia Marina, Universidad Catolica del Norte, Coquimbo, IV Region, Chile

**Keywords:** Mitochondrial genomes, Endangered species, eDNA, Metagenome

## Abstract

The Amur or Siberian tiger *Panthera tigris altaica* (Temminck, 1844) is currently restricted to a small region of its original geographical range in northwestern Asia and is considered ‘endangered’ by the IUCN Red List of Threatened Species. This solitary, territorial, and large top predator is in major need of genomic resources to inform conservation management strategies. This study formally tested if complete mitochondrial genomes of *P. tigris altaica* can be assembled from non-enriched metagenomic libraries generated from scat eDNA samples using the Illumina sequencing platform and open-access bioinformatics pipelines. The mitogenome of *P. tigris altaica* was assembled and circularized using the pipeline GetOrganelle with a coverage ranging from 322.7x to 17.6x in four different scat eDNA samples. A nearly complete mitochondrial genome (101x) was retrieved from a fifth scat eDNA sample. The complete or nearly complete mitochondrial genomes of *P. tigris altaica* were AT-rich and composed of 13 protein coding genes (PCGs), 22 transfer RNA genes, two ribosomal RNA genes, and a putative control region. Synteny observed in all assembled mitogenomes was identical to that reported before for *P. tigris altaica* and other felids. A phylogenomic analysis based on all PCGs demonstrated that the mitochondrial genomes assembled from scat eDNA reliably identify the sequenced samples as belonging to *P. tigris* and distinguished the same samples from closely and distantly related congeneric species. This study demonstrates that it is viable to retrieve accurate whole and nearly complete mitochondrial genomes of *P. tigris altaica* (and probably other felids) from scat eDNA samples without library enrichment protocols and using open-access bioinformatics workflows. This new genomic resource represents a new tool to support conservation strategies (bio-prospecting and bio-monitoring) in this iconic cat.

## Introduction

Global and local environmental changes characteristic of the Anthropocene are driving the rapid loss of biodiversity, including sharp declines in species abundances, particularly large terrestrial vertebrates (Dinerstein et al., 2007; Joshi et al., 2016). Restoring the populations of these large land vertebrates impacted by human activities is one of the central but most challenging goals in wildlife conservation and management (Steffen et al., 2018; Ceballos, Ehrlich & Raven, 2020; Bradshaw et al., 2021). Importantly, many species of land vertebrates for which the scientific community still lack basic biological knowledge, including vulnerable, endangered, and at-risk-of-extinction species, have recently declined (Le Breton et al., 2019). It may be prudent to avoid disturbing declining and sensitive species using invasive bio-prospecting and bio-monitoring techniques; invasive sampling can disturb or stress individuals and populations already subjected to major local anthropogenic impacts (Le Breton et al., 2019).

Environmental DNA (eDNA) has materialized as a reliable substitute to invasive techniques for the sampling of endangered species. Environmental DNA includes the genetic material present in environmental samples such as water, air, soil, sediment, saliva, skin, blood, and feces, among others (Ruppert, Kline & Rahman, 2019). eDNA sampling has been employed to estimate the abundance, genetic diversity, and population structure of elusive species (Bozarth et al., 2010; Wilbert et al., 2019) and has proved useful in situations in which invasive sampling is logistically challenging and ethically fraught (Carøe et al., 2017; Carroll et al., 2018; Parker et al., 2021). Most recently, eDNA retrieved from scats have been used for bio-prospecting (González et al., 2009; Grattarola, Gonzalez & Cosse, 2014) and bio-monitoring (Taberlet et al., 1996; Tian et al., 2021), among other uses (Carøe et al., 2017; Ortega et al., 2004; Seok et al., 2019; Tian et al., 2021). High quality (high molecular weight) genomic DNA isolation from scats still represents a major challenge that limits sequencing and assemblage of complex sequences (*e.g*., bacterial chromosomes or mitochondrial genomes) directly from scats. Recent studies have demonstrated that complete or nearly complete bacterial genomes (Moss, Maghini & Bhatt, 2020), mitochondrial genomes (Matsui et al., 2007; van der Valk et al., 2017), and most recently, nearly complete nuclear genomes (Taylor et al., 2021) can be assembled from scat eDNA. In this study, I formally tested if entire or nearly complete mitochondrial genomes can be retrieved from scat eDNA samples in an iconic endangered species of large cat that has experienced major anthropogenic-driven impact during the last 150 years (Dinerstein et al., 2007).

The Siberian or Amur tiger *P. tigris altaica* is considered one of six extant subspecies of *P. trigris* (Liu et al., 2018) and is currently restricted to a very small fraction of its historic geographic range in China, the Korean Peninsula, and Russia (Dinerstein et al., 2007). *Panthera tigris altaica* is the largest apex predator in the region. Individuals are solitary and highly territorial, with range sizes generally exceeding 30 km^2^. Ranges of viable populations are restricted to forested habitat (Sanderson et al., 2010). During the last century, *P. tigris altaica* has experienced major population declines due to human activities that include but are not limited to landscape conversion (*i.e*., forest loss, urbanization including human settlement and road construction, cattle grazing, cropland development), habitat degradation (including prey depletion), formerly legal hunting, and contemporary poaching, both of which support the underground trade in tiger parts (Dinerstein et al., 2007; Sanderson et al., 2010; Joshi et al., 2016; Wang et al., 2018). The Amur tiger is classified as endangered in the IUCN Red List (Miquelle, Darman & Seryodkin, 2011). Due to the multiple threats facing remaining populations, it is essential to continue advancing non-invasive genomic resources that can help monitor population health and guide the recovery of this iconic endangered felid.

This study formally tested the feasibility of mitochondrial genome *de novo* assembly from WGS generated from fecal DNA. If successful, this genomic tool can be used for bio-monitoring and bio-prospecting as well as to understand population genomics in this iconic endangered cat using relatively inexpensive molecular markers and open-source bioinformatics pipelines.

## Methods

### *Panthera tigris altaica* scat samples

**Figure 1:**
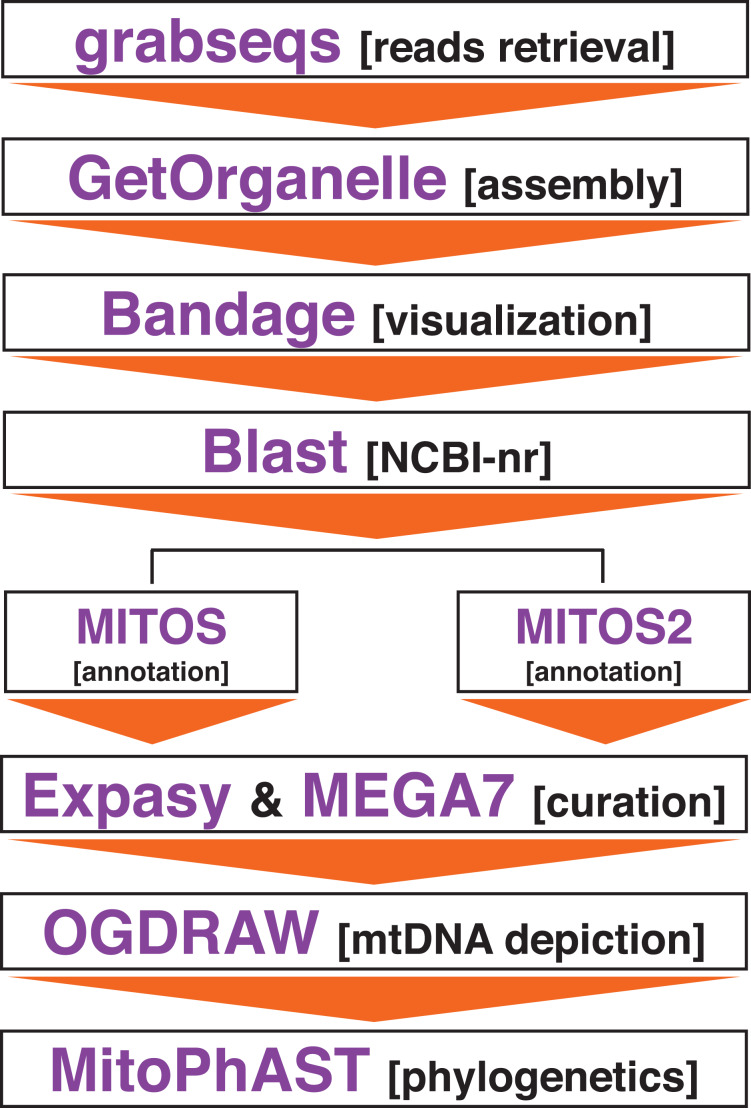
Bioinformatics pipeline to assemble the mitochondrial genome of *Panthera tigris altaica* from scat eDNA samples.

The raw sequence data used to assemble the mitochondrial genome of *P. tigris altaica* from eDNA was generated by He et al. (2018) and employed to describe the effect of two different diets on the gut microbiome of five different captive cubs. In brief, fresh fecal samples from five tiger cubs were collected at a single time point from the Heilongjiang Siberian Tiger Park, Heilongjiang Province, China, Next, genomic DNA (gDNA) was extracted from the fecal samples using the E.Z.N.A.® Stool DNA Kit (D4015–02; Omega, Inc., Norwalk, CT, USA) according to the manufacturer’s instructions. Then, paired-end (PE) shotgun libraries were constructed using a TruSeq Nano DNA LT Library Preparation Kit (FC-121-4001) following the manufacturer’s instructions. Finally, libraries were sequenced in an Illumina sequencer (Illumina, San Diego, CA, USA) using a 2 × 150 cycle. For further details, see He et al. (2018). The five different sets of sequences were retrieved from GenBank (SRA Accession numbers: SRR7429862–SRR7429866) (Fig. 1). The amount of data available in FASTQ format varied between 42,819,376 (SRR7429862) and 78,803,225 (SRR7429864) PE reads per sample (Table 1). All of the available reads generated for each faecal sample was used for mitochondrial genome assembly.

**Table 1:** Parameters for the different mitochondrial genomes assembled from eDNA scat samples in *Panthera tigris altaica*.

Sample	N Read (PE)	Assembly	Length	Coverage*
SRR7429862	42,819,376	Circular	17,071	15.2x/13.9x
SRR7429863	51,488,898	Circular	17,095	17.6x/16.6x
SRR7429864	78,803,225	Circular	17,071	33.5x/32.9x
SRR7429865	59,780,851	Circular	16,862	322.7x/104.8x
SRR7429866	56,765,111	Linear	16,964	101x/118.2x

**Note:**

* The first and second values are coverage estimates from GetOrganelle using the entire mitochondrial genome of *P. tigris altaica* as a ‘seed’ (first run) and only the *cox1* gene (second run), respectively.

### Mitochondrial genome assembly of *Panthera tigris* *altaica* from metagenomic faecal samples

Assembling of the mitochondrial genome from the different scat samples was attempted using the target-restricted-assembly pipeline GetOrganelle v1.2.3 (Jin et al., 2020). GetOrganelle uses a seed-and-extend algorithm that assemble organelles, including mitochondrial genomes, from whole genome sequencing (WGS), including metagenomic datasets, starting from a related or distant single ‘seed’ sequence (Jin et al., 2020) (Fig. 1). For each metagenomics sample, GetOrganelle was run twice. In the first run, the complete mitochondrial genome of *P. tigris altaica* (MH893763, from China) retrieved from GenBank was used as the ‘seed’ while in the second run, only a single gene fragment (*cox1*) from the same mitochondrial genome of *P. tigris altaica* retrieved from GenBank was used as a seed. These two runs per sample permitted us to explore the effect of seed length on the quality (*i.e*., completeness) and coverage of the retrieved mitochondrial genome assemblies. All the different runs used k-mer sizes of 21, 55, 85, and 115. Reads were not quality trimmed prior to assembly using the GetOrganelle pipeline following the developer’s guidelines (Jin et al., 2020). The software Bandage (Wick et al., 2015) was used to visualize the assembly graph generated by the pipeline above and any assembled contigs were compared to the nucleotide non-redundant database in NCBI’s GenBank in order to determine if these contigs belonged to the mitochondrial genome of the target species. I predicted that a circularized (or not) sequence ~17 kpb in length would be observed among the contigs if the pipeline above successfully assembled the mitochondrial genome of *P. tigris altaica*.

### Annotation and analysis of mitochondrial genome or contigs

Complete or partially assembled mitochondrial genomes were first annotated *in-silico* using the web servers MITOS (http://mitos.bioinf.uni-leipzig.de) and MITOS2 (http://mitos2.bioinf.uni-leipzig.de) (Bernt et al., 2013) with the vertebrate genetic code (code 2). Manual curation of the *in-silico* annotations, including start + stop codons corrections, were performed using the web server ExPASy (https://web.expasy.org/) and the software MEGA7 (Kumar, Stecher & Tamura, 2016). Genome visualization was performed in OGDRAW–Draw Organelle Genome Maps (https://chlorobox.mpimp-golm.mpg.de/OGDraw.html) (Lohse, Drechsel & Bock, 2007) (Fig. 1).

### Phylogenetic position of mitochondrial genomes retrieved from feces

The phylogenetic position of the mitochondrial genomes assembled from scat eDNA was examined among other representatives belonging to the genus *Panthera*. The five newly assembled and annotated mitochondrial genomes of *P. tigris altaica* plus those (complete or nearly complete) of other 67 specimens of *Panthera*, all of them available in the GenBank database (consulted 11 07 2021), were used for the phylogenetic analysis conducted with the program MitoPhAST (Tan et al., 2015). One mitochondrial genome belonging to the cloud leopard *Neofelis nebulosi* was used as an outgroup. In MitoPhAST, all 13 protein-coding gene nucleotide sequences from the species used in this analysis were retrieved from GenBank files, translated to amino acids, and aligned using Clustal Omega (Sievers et al., 2011). Next, poorly aligned regions with large numbers of indels were removed with trimAl (Capella-Gutierrez, Silla-Martinez & Gabaldon, 2009). Partitions and best fitting models of sequence evolution for each partition were selected with ProtTest (Abascal, Zardoya & Posada, 2005). The concatenated and partitioned protein-coding gene amino acid alignments were used to perform a maximum likelihood phylogenetic analysis in the software IQ-TREE (Nguyen et al., 2015). The robustness of the ML tree topology was assessed by bootstrapping the observed data 1,000 times. In this study, we used amino acids instead of nucleotide characters for phylogenetic inference considering that the former markers have a greater ratio of phylogenetic information to noise, compared to nucleotides, for resolving basal and derived nodes in a phylogenetic tree (Page & Holmes, 2009).

## Results and discussion

### Assembly of mitochondrial genomes from *Panthera trigis altaica* field scats

The program GetOrganelle assembled and circularized the mitochondrial genome of *P. tigris altaica* in four of the five tested scat eDNA metagenomics samples. Furthermore, the two strategies (full mitochondrial genome vs only *cox1* seed) employed to assemble the mitochondrial genome of *P. tigris altaica* resulted in identical sequences in each scat eDNA sample. The length and coverage of these circularized mitochondrial genomes varied between 16,862 bp (coverage = 322.7x per nucleotide) and 17,095 bp (17.6x) in samples SRR7429865 and SRR7429863, respectively. In one sample (SRR7429866), Getorganelle assembled but did not circularize the mitochondrial genome of *P. tigris altaica*. The length and coverage of this assembled nearly complete mitochondrial genome was 16,964 bp and 101x, respectively (Table 1). Importantly, this assembled mitochondrial genome was only 16 bp shorter compared to the reference genome used for its assembly (MH893763).

### Annotation of mitochondrial genomes assembled from scats of *Panthera trigis altaica*

**Figure 2:**
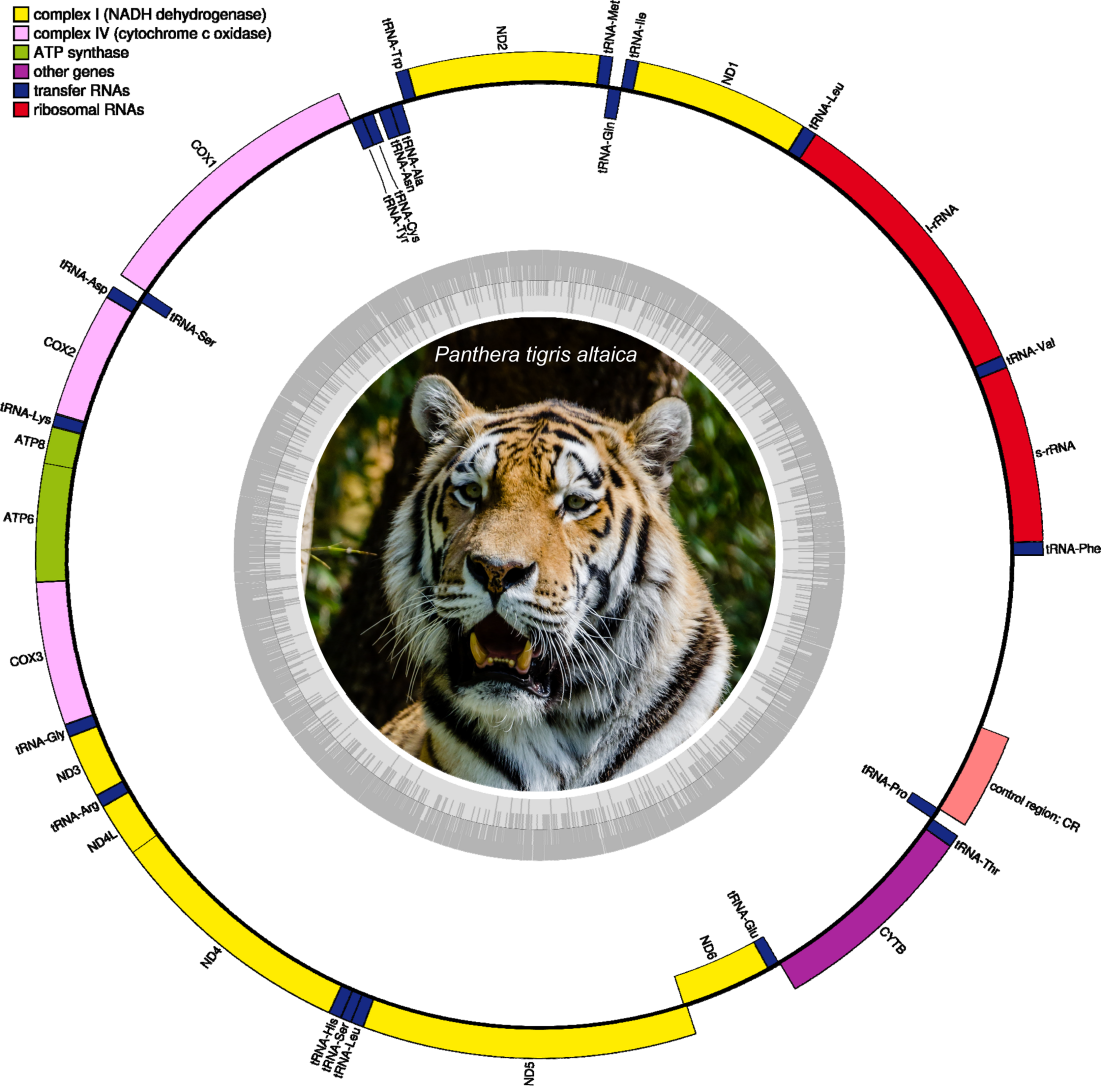
Circular DNA mitochondrial genome map of *Panthera tigris* *altaica* assembled from eDNA scat (sample SRR7429863). The annotated map depicts 13 protein-coding genes (PCGs), two ribosomal RNA genes (rrnS: 12 S ribosomal RNA and rrnL: 16 S ribosomal RNA), 22 transfer RNA (tRNA) genes, and the putative control region (not annotated). Photo credit: Tuxyso/Wikimedia Commons, CC BY-SA 3.0. https://commons.wikimedia.org/w/index.php?curid=22692001.

All mitochondrial genome assemblies, either complete or partial, of *Panthera tigris altaica* retrieved from scat eDNA samples were composed of 13 protein-coding genes, two ribosomal RNA genes (rrnS (12S ribosomal RNA) and rrnL (16S ribosomal RNA)), 22 transfer RNA (tRNA) genes, and a non-coding region of variable length (Fig. 2, Table S1). Most of the protein-coding and tRNA genes were encoded on the L-strand, while only a single protein-coding gene (*nad6*) and eight tRNA genes (5′ to 3′ order: *trnQ, trnA, trnN, trnC, trnY, trnS2, trnE, trnP*) were encoded in the H-strand (Fig. 2, Table S1). The gene order observed in these complete or nearly complete mitochondrial genomes was identical to that reported before in *Panthera tigris altaica* as well as the whole genus *Panthera* (Wei, Wu & Jiang, 2009; Kitpipit & Linacre, 2012; Dou et al., 2016). A detailed characterization of the features present in all complete mitochondrial genomes is provided below in order to explore the accuracy of these assemblies retrieved from scat eDNA samples.

Nucleotide usage (overall % base composition) of the mitochondrial genomes assembled from the different scat samples (SRR7429862–SRR7429866) is presented in Table 2. All assembled mitochondrial genomes were compact with only a few intergenic spaces and overlaps among gene junctions (Table S1). In all circularized mitochondrial genomes, a single relatively long intergenic space varying in length between 1,413 bp in sample SRR7429865 to 1,531 bp in sample SRR7429863 was assumed to be the Control Region/D-loop that is involved in replication of this genome (Bernt et al., 2013).

**Table 2:** Nucleotide usage of the complete mitochondrial genome (and genes) assembled from eDNA scat samples in *Panthera tigris altaica*.

Sample	A%	T%	C%	G%
mtDNA				
SRR7429862	31.81	26.99	26.58	14.63
SRR7429863	31.81	26.97	26.60	14.62
SRR7429864	31.81	26.99	26.58	14.63
SRR7429865	31.78	24.04	26.54	14.63
SRR7429866	31.83	26.98	26.57	14.61
rrnS gene				
SRR7429862	35.76	22.25	23.28	18.19
SRR7429863	35.76	22.25	23.80	18.19
SRR7429864	35.76	22.25	23.80	18.19
SRR7429865	35.76	22.25	23.80	18.19
SRR7429866	35.86	22.25	23.80	18.09
rrnL gene				
SRR7429862	36.91	23.44	21.66	17.98
SRR7429863	36.91	23.44	21.66	17.98
SRR7429864	36.91	23.44	21.66	17.98
SRR7429865	36.91	23.44	21.66	17.98
SRR7429866	36.79	23.44	21.66	18.11

In all the complete mitochondrial genome assemblies retrieved from scat eDNA samples, all of the 13 PCGs exhibited conventional vertebrate mitochondrial start codons; ATA, ATC, and ATG (Table 1). Invariably, eleven PCGs terminated with a complete and conventional stop codon (TAA or TAG) while the genes *nad4* and *cox3* terminated with the incomplete stop codons T and TA, respectively. Truncated stop codons are thought to be completed *via* post-transcriptional poly-adenylation (Ojala, Montoya & Attardi, 1981; Rorbach & Minczuk, 2012 and references therein).

In the retrieved mitochondrial genome assemblies of *P. tigris altaica*, the most frequently used codons found in the PCGs were AT-rich, and included CTA (Leu, *n* = 241–242 in 3 samples, 270 in one sample), ATA (Met, *n* = 176 in all samples), and ATC (Ile, *n* = 170–171 in all samples). In turn, discounting stop codons, least frequently used codons were GC-rich, and included CGG (Arg, *n* = 3 in all samples), CCG (Gln, *n* = 7–8 in all samples), GCG (n = 7 in all samples), and CGT (n = 7 in all samples). This aforementioned codon usage pattern is akin to that reported before for protein-coding genes in the mitochondrial genome of *P. tigris altaica* and other congeneric species whose codon usage pattern has been examined (Wei, Wu & Jiang, 2009; Kitpipit & Linacre, 2012; Dou et al., 2016).

In each one of the mitochondrial genome assemblies, tRNA genes ranged in length from 59 (*trnS1*) to 75 bp (*trnL2*) (Table S1). All but one tRNA gene (*trnS1*) exhibited a standard ‘cloverleaf’ secondary structure. In the *trnS1* gene of all assemblies, the stem and loop of the pseudouridine arm (T-arm) was missing. This truncated *trnS1* represents a conserved trait among vertebrates and eumetazoans (Bernt et al., 2013) and has also been found in all other representatives of the family Felidae in which the secondary structure of tRNA genes have been examined (Wei, Wu & Jiang, 2009; Kitpipit & Linacre, 2012; Dou et al., 2016). Whether or not truncated tRNAs interact with other molecular factors while decoding mRNA into protein remains to be investigated in species belonging to the family Felidae as well as in many other vertebrates and invertebrates (Watanabe, Suematsu & Ohtsuki, 2014).

In all assemblies, the length of the rrnS (12S) and rrnL (16S) genes was 962 and 1,574 bp, respectively. These two ribosomal genes were located close to each other; the rrnS gene was located between the trnF and trnV genes while the rrnL gene was located between the trnV and trnL2 genes. The two genes exhibited an AT-skew. Nucleotide usage of the rrnS and rrnL genes are presented in Table 2.

Overall, the annotation and detailed characterization above indicates that whole or nearly complete mitochondrial genome assemblies retrieved from eDNA are accurate.

### Phylogenetic position of mitochondrial genomes retrieved from feces

**Figure 3:**
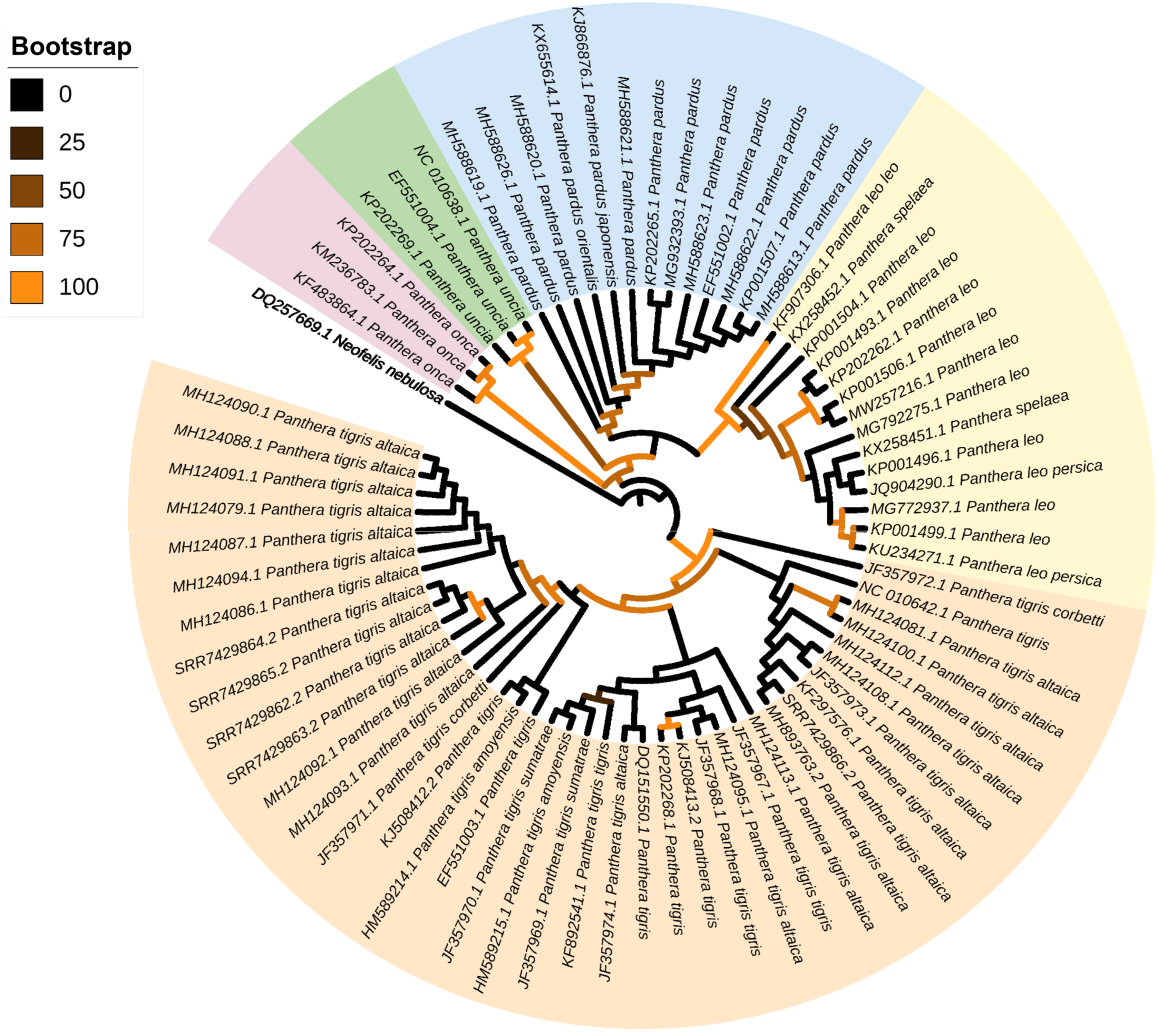
Mitophylogenomics of the genus *Panthera*. Phylogenetic analysis of *Panthera tigris altaica* scat samples, conspecifics, and related congeneric species in the subfamily Pantherinae. Total evidence phylogenetic tree obtained from ML analysis based on a concatenated alignment of amino acids of the 13 protein-coding genes present in the mitochondrial genome of representatives of the subfamily Pantherinae. In the analysis, one species of the subfamily Felinae was used as outgroup. Numbers above or below the branches represent bootstrap values.

The ML phylogenetic tree (terminals: 73, amino acid characters: 3,792, informative sites: 286) indicated monophyly of the genus *Panthera* with high support values (bv = 100) (Fig. 3). Within the genus *Panthera*, all representatives of *P. onca* (*n* = 3 terminals) clustered into a single well supported clade (bv = 97) that was sister to all other specimens used in the analysis. In the tree, the five mitochondrial genomes assembled from *P. tigris altaica* scat eDNA together with other 34 complete or nearly complete mitogenomes of the same species *P. trigris* retrieved from Genbank clustered into a very well supported clade (bootstrap value [bc] = 98). Within this monophyletic *P. trigris* clade, the different specimens did not segregate according to subspecies (Fig. 3). This is not necessarily unexpected considering that ancient hybridization events among species and historic admixture and gene flow between isolated populations are apparently widespread in felids, including within the genus *Panthera* (de Manuel et al., 2020). The other well-supported clade (bv = 99) was composed of all representatives belonging to *P. leo* and the extinct cave lion *P*. *spelea*. Within this *P. leo* + *P*. *spelea* clade, the different specimens did not segregate according to species, in contrast to a previous study that relied on a smaller number of mitochondrial genomes to reveal the phylogenetic position of the cave lion (Barnett et al., 2016). Two moderately-supported clades included those formed by all specimens belonging to *P. uncia* (bv = 59) and *P. pardus* (bv = 69). The latter clade was sister to the well-supported clade composed of *P. leo* + *P*. *spelea* (Fig. 2). Overall, this analysis demonstrates that the mitochondrial genomes assembled from eDNA scat samples can and do reliably identify specimens from which scat samples were obtained as belonging to *P. tigris*. Furthermore, our analysis shows that mitochondrial genomes assembled from scat samples can be distinguished from closely related congeners.

Our mitogenomic *Panthera* phylogeny differs from the most recent *Panthera* phylogeny estimated from genome-wide data (Figueiró et al., 2017). In contrast to that observed in our analysis, Figueiró et al. (2017) placed *P. tigris* as sister to *P. uncia*, as well as *P. leo* and the jaguar *P. onca* sister to *P. pardus*. The contrast in topologies based on mitochondrial genomes alone (this study) *vs*. genome-wide markers might be due to ancient hybridization events and historic admixture (de Manuel et al., 2020).

## Conclusions

This study was the first to assemble mitochondrial genomes belonging to the endangered Siberian tiger *P. tigris altaica* from scat eDNA metagenomic libraries. Our bioinformatics workflow presents a new tool in conservation biology developed for the endangered *P. tigris altaica*; a similar workflow can be applied to build whole mitochondrial genomes from eDNA samples of other species. Further development of genome assembly from scat eDNA samples will contribute to identification and monitoring of *P. tigris altaica* as well as other large carnivores. The assembly of complete mitochondrial genomes from eDNA scat samples can improve our understanding of population density, deme dynamics, and the presence/absence of this species in human altered habitats. The author propose this newly assembled genome can be used as a reference for the retrieval of mitochondrial markers of *P. tigris altaica* when using indirect surveillance strategies such as scat eDNA (Schnell et al., 2015). Such efforts are currently being tested in other large vertebrates with major conservation problems (*e.g*., in moose–Lyet et al., 2021) and this study is a step forward towards to implementation of indirect field surveillance in *P. tigris altaica*. Considering that the complete removal of fecal samples from the field might increase agonistic interactions among individuals in territorial species such as tigers, the author suggest the partial removal and sampling of scat from the wild (see Lefort et al., 2022).

Ultimately, our understanding of population genomics and genomic responses of large carnivores to anthropogenic impacts will increase with the further development of complete mitochondrial and nuclear genomes of threatened and rare species.

## Supplemental Information

10.7717/peerj.14428/supp-1 Supplemental Information 1 Mitochondrial genomes assembled from scat eDNA in *Panthera tigris* altaica. Arrangement and annotation.

## Additional Information and Declarations

### Data Availability

The following information was supplied regarding data availability: The five different sets of sequences are available at GenBank: SRR7429862–SRR7429866. The five different mitochondrial genomes assembled from eDNA scat samples are available at GenBank: BK062820-BK062824.
