# Supplementary material for: Mitochondrial genomes assembled from non-invasive eDNA metagenomic scat samples in the endangered Amur tiger Panthera tigris altaica
Source: PeerJ. 2022 Dec 6;10:e14428. doi: 10.7717/peerj.14428 (PMC9745948; doi:10.7717/peerj.14428)
Supplement: Supplemental Information 1 — Arrangement and annotation. [file peerj-10-14428-s001.doc]

**Table S1**. Mitochondrial genomes assembled from scat eDNA in *Panthera tigris altaica.* Arrangement and annotation.

**Sample SRR7429862**

**Sample SRR7429863**

**Sample SRR7429864**

**Sample SRR7429865**

**Sample SRR7429866**
